# Bradycardia, Renal Failure, Atrioventricular Nodal Blockade, Shock, and Hyperkalemia (BRASH) Syndrome Triggered by Missed Dialysis: A Case Report

**DOI:** 10.7759/cureus.83713

**Published:** 2025-05-08

**Authors:** Kesava Manikanta Achuta, Venkata Vedantam, Sai Karthik Kommineni, Neethu Vedantam, Kyasa S Venisha

**Affiliations:** 1 Internal Medicine, Garden City Hospital, Michigan State University, Dearborn Heights, USA; 2 Internal Medicine, East Tennessee State University Quillen College of Medicine, Johnson City, USA; 3 Infectious Diseases, East Tennessee State University Quillen College of Medicine, Johnson City, USA; 4 Radiology, Aditya Diagnostics, Godavarikhani, IND

**Keywords:** bradycardia, brash syndrome, end-stage renal disease, hyperkalemia, renal dysfunction

## Abstract

Bradycardia, renal failure, atrioventricular (AV) nodal blocker usage, shock, and hyperkalemia are the hallmarks of BRASH syndrome, a complex medical emergency. Despite having a high mortality rate, BRASH syndrome is underdiagnosed and frequently develops from the combined effects of renal failure and drug toxicity. Here, we present the case of an 86-year-old woman with end-stage renal disease (ESRD) who developed BRASH syndrome due to missed dialysis sessions, exacerbated by low-dose beta-blocker therapy. This case emphasizes how crucial it is to identify BRASH syndrome early, treat it quickly, and address its untoward events to avoid fatalities.

## Introduction

BRASH syndrome is a constellation of bradycardia, renal failure, atrioventricular (AV) nodal blocker use, shock, and hyperkalemia [1]. This syndrome arises from the synergistic effects of hyperkalemia and renal impairment, which amplify the pharmacologic action of AV nodal blockers, leading to severe bradycardia and conduction abnormalities. It predominantly affects individuals with poor renal functional reserve and preexisting kidney dysfunction.

The syndrome can be triggered by a range of factors, including hypovolemia, recent adjustments of antihypertensive medications, or concomitant use of agents such as angiotensin-converting enzyme (ACE) inhibitors, angiotensin receptor blockers (ARBs), aldosterone receptor antagonists, and calcium channel blockers [1]. Chronic kidney disease and hyperkalemia are common predisposing factors. Clinically, patients present with a wide spectrum of symptoms, ranging from asymptomatic bradycardia, fatigue, and lethargy to syncope and severe cardiogenic shock.

This report discusses an 86-year-old woman with end-stage renal disease (ESRD) who developed hemodynamic instability and shock secondary to BRASH syndrome following missed dialysis sessions. This case highlights the importance of early identification and prompt treatment and addresses important factors to prevent fatal outcomes.

## Case presentation

An 86-year-old woman with a medical history significant for ESRD on hemodialysis, hypertension, atrial fibrillation, dementia, prior stroke, hypothyroidism, and depression was brought to the emergency department (ED) from her nursing home due to lethargy and minimal responsiveness that had progressed over several days. Initially, her confusion was attributed to delirium secondary to dementia, but her rapid clinical deterioration prompted her family to seek emergency care. Upon arrival in the ED, the patient was found to be lethargic and somnolent. The family reported that the patient had missed her hemodialysis sessions the previous week and had experienced progressive lethargy, decreased oral intake, and worsening confusion since then. She had no prior history of bradycardia or significant bleeding manifestations such as melena, hematochezia, hematemesis, or falls/head injury. Her home medications include apixaban 2.5 mg BID, metoprolol succinate 25 mg daily, isosorbide 30 mg daily, and hydralazine 37.5 mg thrice daily, as well as sevelamer 800 mg thrice daily and levothyroxine 25 mcg daily. Her baseline hemoglobin was 8.5 g/dL, consistent with anemia of chronic kidney disease. At presentation, her vital signs included a blood pressure of 78/46 mmHg, a heart rate of 31 beats/minute, and an SpO2 of 96% on room air, and the patient was afebrile. On examination, the patient was lethargic, with bilateral basal lung crackles, lower extremity edema, and weak peripheral pulses. The laboratory findings are shown in Table 1.

**Table 1:** Laboratory findings WBC: white blood cell count, BUN: blood urea nitrogen

Laboratory parameters	On admission	On discharge	Reference value
Hemoglobin	6.9 g/dL	8.2 g/dL	12-15.5 g/dL
WBC	6,600 cells/µL	7,000 cells/µL	3,500-10,000 cells/µL
Platelets	128,000 cells/µL	160,000 cells/µL	150,000-450, 000cells/µL
Sodium	136 mmol/L	138 mmol/L	136-145 mmol/L
Potassium	7.2 mmol/L	4.1 mmol/L	3.5-5.3 mmol/L
Calcium	7.5 mg/dL	8.4 mg/dL	8.6-10.4 mg/dL
Phosphorous	8.7 mg/dL	4.2 mg/dL	2.1-4.3 mg/dL
BUN	117 mg/dL	47 mg/dL	6-24 mg/dL
Creatinine	8.59 mg/dL	4.33 mg/dL	0.5-1 mg/dL
Bicarbonate	20 mmol/L	29 mmol/L	20-31 mmol/L
Lactic acid	0.9 mmol/L	-	0-2 mmol/L
Troponin I	82 ng/L	-	<34 ng/L

**Figure 1:**
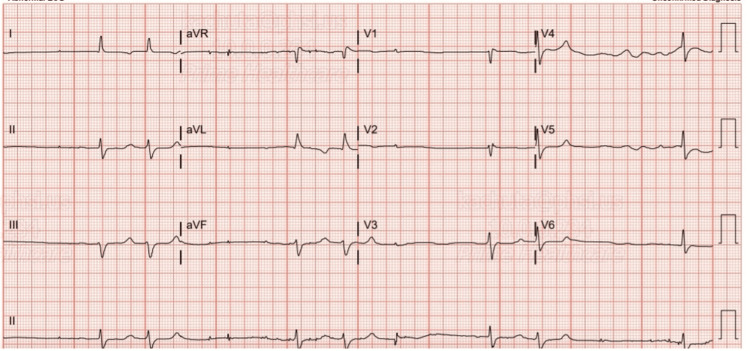
ECG after administration of atropine ECG: electrocardiography

Electrocardiography (ECG) showed an irregularly irregular rhythm with a heart rate of 31 bpm, consistent with atrial fibrillation. After administration of 0.4 mg atropine, the patient's heart rate improved to 59 bpm, as can be seen in Figure 1, and her blood pressure stabilized at 90/60 mmHg. A transthoracic echocardiogram revealed a normal ejection fraction of 55%-60%, with no evidence of structural abnormalities or impaired cardiac function.

The patient received calcium gluconate to stabilize cardiac membranes, intravenous insulin with dextrose to promote intracellular potassium shift, sodium zirconium cyclosilicate to facilitate potassium elimination, and emergent hemodialysis with a 1K potassium bath to correct hyperkalemia and uremia. She also received one unit of packed red blood cells to address anemia.

Follow-up laboratory results showed marked improvement. Her hemoglobin rose to 9 g/dL, potassium normalized to 4 mmol/L, and creatinine decreased to 4.6 mg/dL after hemodialysis. With hemodynamic stability restored, the patient was transferred to a step-down unit for continued monitoring. Sepsis was initially considered due to hypotension, bradycardia, renal dysfunction, and a poorly maintained perm catheter. However, the absence of fever, leukocytosis, or elevated lactic acid, along with negative follow-up cultures, suggested contamination rather than true infection.

Before discharge, the patient and her family received comprehensive education on the importance of dialysis adherence, medication compliance, and the avoidance of nephrotoxic agents. She was also counseled on recognizing early signs of BRASH syndrome to prevent recurrence. The patient was discharged home in stable condition with appropriate follow-up care.

## Discussion

BRASH syndrome results from the synergistic effect of hyperkalemia and AV nodal blockers (e.g., beta-blockers and calcium channel blockers) in the presence of precipitating factors, usually renal impairment (acute kidney injury or acute-on-chronic kidney disease), hypovolemia, or medications such as ACE inhibitors/ARBs that can cause both hyperkalemia and renal dysfunction. In our case, the combination of hyperkalemia and reduced renal clearance of AV blockers due to dialysis non-compliance created a vicious cycle. Hyperkalemia worsened bradycardia, leading to hypotension, shock, and further impairment of renal perfusion. Without timely intervention, this cycle can rapidly progress to a potentially fatal outcome. Elderly patients are particularly vulnerable due to age-related sinus node dysfunction, lower resting heart rates, and polypharmacy. The average age of presentation is approximately 69 years [2]. Although the exact prevalence is unknown, BRASH syndrome is neither exceedingly rare nor uncommon. While BRASH syndrome is more common with larger doses of beta-blockers or extended-release formulations, cases have been reported with doses as low as 50 mg of metoprolol succinate. Remarkably, our patient exhibited the spectrum of BRASH syndrome with just 25 mg of metoprolol succinate. This underscores the importance for physicians to remain vigilant and aware of the potential for the occurrence of BRASH syndrome even with low doses of beta-blockers [3].

**Figure 2:**
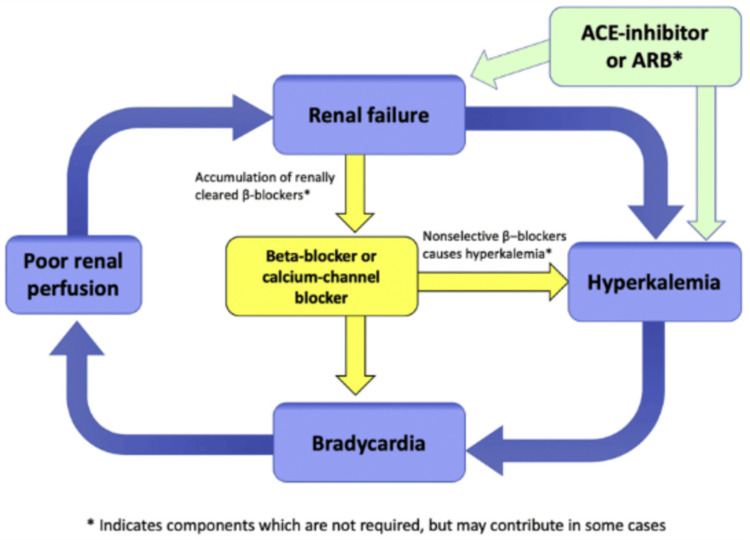
Pathophysiology of BRASH syndrome This image is adapted from Farkas JD, Long B, Koyfman A, Menson K: BRASH syndrome: bradycardia, renal failure, AV blockade, shock, and hyperkalemia. J Emerg Med. 2020, 59:216-23. 10.1016/j.jemermed.2020.05.001 [4], licensed under CC BY-NC-ND 4.0. Permission was obtained from the author/publisher. BRASH: bradycardia, renal failure, AV blockade, shock, and hyperkalemia, ACE: angiotensin-converting enzyme, ARB: angiotensin receptor blocker

Recognizing the constellation of findings and addressing all components of BRASH syndrome is critical. A common pitfall is focusing on a single issue, such as hyperkalemia, while neglecting other contributors, such as bradycardia or renal dysfunction. Effective management requires an integrated approach to stabilize all components of the syndrome [4]. Electrocardiography (ECG) plays a key role in diagnosis, particularly in differentiating BRASH syndrome from isolated hyperkalemia. Hyperkalemia alone typically presents with tall T waves, PR interval prolongation, and QRS widening. In contrast, our patient's ECG lacked these features, indicating a more complex interplay of factors [4]. Figure 2 depicts the BRASH syndrome pathophysiology [4].

Failure to recognize BRASH syndrome can lead to unnecessary interventions and mismanagement. For instance, unwarranted pacemaker implantation for reversible bradycardia is a known risk. A study in Philadelphia County reported that up to 20% of pacemakers were implanted without clear indications, increasing the risk of complications such as infections and potential mortality [5]. Similarly, focusing solely on correcting electrolytes or treating bradycardia in isolation may increase mortality by failing to address the underlying pathophysiology [6]. Given that over one million pacemakers are implanted annually worldwide, accurate identification of reversible causes such as BRASH syndrome should take precedence over preemptive device placement [7].

The management of BRASH syndrome involves addressing hyperkalemia, bradycardia, renal dysfunction, and any contributory hypovolemia. Bradycardia is treated with agents such as atropine, isoproterenol, or epinephrine, and AV nodal blockers are temporarily discontinued or substituted until the acute condition resolves. Notably, Advanced Cardiovascular Life Support (ACLS) guidelines for bradycardia may not fully apply in BRASH syndrome [8]. Hyperkalemia is managed with calcium gluconate for myocardial stabilization, insulin with dextrose for potassium shifting, and, if needed, emergency hemodialysis with low-potassium baths. Renal dysfunction is treated by addressing its underlying cause, such as fluid resuscitation for hypovolemia, antibiotics and fluids for sepsis, or emergent dialysis for missed sessions, as in this case. If AV nodal blocker toxicity contributes to the syndrome, high-dose insulin euglycemia therapy, glucagon, or lipid emulsions may be used for lipid-soluble drugs.

Permanent pacemaker placement is rarely required, as bradycardia in BRASH syndrome is generally reversible with medical management. Temporary transcutaneous pacing may be required in refractory cases and has been documented in up to 32.9% of patients. In our patient, bradycardia resolved with one dose of atropine and a session of hemodialysis, which corrected both hyperkalemia and renal dysfunction.

The decision to resume AV nodal blockers after BRASH syndrome is not well defined and typically involves shared decision-making between the physician and the patient, weighing the risks and benefits. In this case, AV blockers were resumed post-discharge for rate control of atrial fibrillation. Social services were engaged to address the barriers to regular dialysis, mitigating the risk of recurrence [9].

## Conclusions

This case contributes to the medical literature by highlighting the importance of recognizing BRASH syndrome as a complex, multifactorial condition, emphasizing the need for comprehensive management that addresses hyperkalemia, bradycardia, and renal dysfunction simultaneously. It further highlights the occurrence of BRASH syndrome even with low doses of AV nodal blockers, underscoring the importance of timely recognition and comprehensive management. By emphasizing the interplay of hyperkalemia, bradycardia, and renal dysfunction, it raises awareness of this under-recognized yet potentially life-threatening condition. The report also stresses the need to address precipitating factors, such as missed dialysis, to prevent recurrence and demonstrates that invasive interventions such as pacemaker placement are rarely required when timely medical therapy is provided, thus providing essential guidance for clinicians to adopt a holistic approach to optimize treatment strategies and improve patient outcomes.
